# Effect of Foot Orthoses and Shoes in Parkinson’s Disease Patients: A PRISMA Systematic Review

**DOI:** 10.3390/jpm11111136

**Published:** 2021-11-02

**Authors:** María Reina-Bueno, César Calvo-Lobo, Daniel López-López, Patricia Palomo-López, Ricardo Becerro-de-Bengoa-Vallejo, Marta Elena Losa-Iglesias, Carlos Romero-Morales, Emmanuel Navarro-Flores

**Affiliations:** 1 Faculty of Nursing, Physiotherapy and Podiatry, University of Sevilla, 41009 Seville, Spain; mreina1@us.es; 2 Faculty of Nursing, Physiotherapy and Podiatry, Complutense University of Madrid, 28040 Madrid, Spain; cescalvo@ucm.es (C.C.-L.); ribebeva@ucm.es (R.B.-d.-B.-V.); 3 Research, Health and Podiatry Group, Department of Health Sciences, Faculty of Nursing and Podiatry, Universidade da Coruña, 15403 Ferrol, Spain; daniellopez@udc.es; 4 University Center of Plasencia, Universidad de Extremadura, 10600 Plasencia, Spain; patibiom@unex.es; 5 Faculty of Health Sciences, Universidad Rey Juan Carlos, 28922 Alcorcón, Spain; marta.losa@urjc.es; 6 Faculty of Sport Sciences, Universidad Europea de Madrid, 28670 Villaviciosa de Odón, Spain; 7 Frailty Research Organized Group (FROG), Department of Nursing, Faculty of Nursing and Podiatry, University of Valencia, 46010 Valencia, Spain; emmanuel.navarro@uv.es

**Keywords:** insole, Parkinson’s disease, footwear, balance, gait

## Abstract

Reduced plantar foot sensation, postural instability, and gait difficulties are characteristic of Parkinson´s disease patients. A systematic review was carried out to determine the effect of the different types of insoles and shoes in these patients. Several databases were used to search for relevant articles reporting Parkinson´s disease patients undergoing treatment with any type of insole and footwear. All titles and abstracts were reviewed independently by two reviewers and the available data were extracted. The study eligibility criteria were any type of experimental study that included Parkinson’s disease patients treated with any type of insole or footwear. Eight studies were selected. Interventions used were textured insoles, footwear modifications, and habitual footwear. Three different outcomes were evaluated in each study: gait parameters, balance, and plantar sensation. According to the data available from this systematic review, the most important conclusion is that more controlled studies are needed in this research field. There are indications to suggest that textured insoles have positive effects on gait parameters, balance, and plantar sensation in Parkinson’s disease patients.

## 1. Introduction

Failure of the dopaminergic cells in the substantia nigra pars compacta is the main pathology in Parkinson’s disease (PD), causing a reduced dopamine level in the basal ganglia [1]. PD results in typical motor abnormalities, from drive to gait impairment and postural instability. The main characteristics of abnormal gait in PD are a slow walking speed, increased stride variability, and short shuffling steps [2].

An anomaly related to the striatum (caudate and putamen) can result in “striatal deformity”—atypical postures of the hand, spine or foot [3]. These deformities may be responsive to levodopa and may be part of the process of the wearing off of dystonia. Misdiagnosis of these deformities as rheumatoid or as some other type of arthritis is very common. Initially these deformities may be flexible, but later they become rigid [4].

The hands and/or feet joints can develop rheumatoid-like deformities; these Parkinsonian signs are poorly known and unrecognized. The types of striatal foot involve equinovarus foot positioning, flexion of the other toes, and great toe extension. Foot deformities disturb gait and stability and produce supplementary disability in patients, adding to the complexity of PD symptoms. Deformities can also produce pain, distress cramp, muscle tension, and may impair the ability to wear shoes. If untreated, skin ulceration, contractures, abnormal gait patterns, and bone erosion may occur [5].

The degeneration of the peripheral sensory nerves and cutaneous receptors can reduce peripheral sensation in people with PD [6]. Abnormal kinesthesia and proprioception contribute to the Parkinsonian gait, even though PD is mainly a motor disease, adding evidence suggesting that PD patients have impaired joint position sense and reduced plantar foot sensation, movement accuracy, and movement perception. A defective proprioceptive feedback and an inadequate integration of sensory inputs induce the abnormal motor control movement in PD [7].

These gait disorders severely restrict patients’ quality of life and mobility and augment the risk of falling [8,9]. The link of falls with femoral fractures, increased dependency, social isolation, and lowered quality of life because of the fear of falling explains their importance [10].

Striatal limb deformities develop in approximately 13% of patients with PD [5]. Reduced plantar foot sensation, postural instability, and gait difficulties are characteristic of PD patients [11]. The use of footwear in these patients reduces peripheral feedback from the feet and restricts foot structure. It has a negative influence on foot functionality [9]. Footwear is a modifiable risk factor in falls prevention. Gait patterns can be altered by footwear [12]. Although pharmacological intervention is the pillar of Parkinson’s treatment, there is weak evidence to indicate that drug therapy reliably increases balance to reduce falls [10].

Therefore, we performed a systematic review to determine how different footwear-based interventions (textured insoles, footwear modifications, and habitual footwear) affect the outcome (gait parameter, balance, and plantar sensation) in PD patients.

## 2. Materials and Methods

### 2.1. Identification, Eligibility Criteria, and Selection of Studies

This systematic review was registered in PROSPERO. The title decided on was “Effect of foot orthoses and shoes in Parkinson’s disease patients”, and the registration number assigned was CRD42020209824. The search took place in the following databases: MEDLINE, Dialnet, Scopus, Web of Science, and The Cochrane Library.

The searches were performed in October 2020, after constructing the strategies to be used in the different databases and after developing a succession of pilot tests to verify the exact implementation of the procedure in each of them. The search strategy carried out can be checked in the Supplementary Materials.

The descriptors foot orthoses, insoles, shoes, and footwear were used, combined with AND Parkinson. The descriptors relating to the terms of relevance were used with CINAHL, PubMed, and The Cochrane Library databases, as well as with the free terms. In all of these, an advanced search tool was employed to create the strategy.

Initially, only randomized controlled clinical trials (RCTs) written in English language and up to 10 years old were selected, but due to the limited number of studies found, the selection criteria were broadened. Neither the language of the documents nor the year of publication were restricted. The inclusion criteria were any type of experimental study, whether RCT, non-randomized controlled clinical trial, non-controlled clinical trial or crossover clinical trial) with Parkinson´s patients of any age, sex, race, or ethnicity. If one of them included the application of any type of insole and/or use of any type of footwear, any kind of comparison between interventions was acceptable. The use of additional physical treatment, orthotics, and/or complementary pharmacological treatment was not a cause for exclusion. Any kind of result was valid.

### 2.2. Assessment of Characteristics of Studies

Two independent reviewers (M.R.B. and E.N.F.) developed the selection procedure. Each evaluator read the title and abstract of each of the documents; independently, they assessed whether each of studies met the inclusion criteria. Independently each reviewer assessed the main characteristics of the studies, indicating whether they matched the eligibility criteria.

Thus, after the first negative item showed, each reviewer could reject the document without the requirement of continuing to assess the rest of it.

The risk of bias evaluation in the studies was carried out using the Review Manager tool (RevMan) of The Cochrane Library, v.5.3. Once obtained, a complete reading of the documents chosen by each of the reviewers was performed, again separately. In the complete reading, we again evaluated its suitability according to precise compliance with the inclusion criteria defined, which was required for the study to be finally chosen for review.

### 2.3. Data Extraction

To simplify the first selection procedure, due to the high number of references analyzed, an Excel page was designed as a data collection form in which the inclusion or non-inclusion of the document and the reason for its elimination were recorded via a codification of the items (criteria). Each reviewer processed his/her own page.

After the first evaluation of the reports, their results were combined, and the inclusion or exclusion of the contradictory documents was debated. The inclusion or exclusion was debated by the two initial reviewers, but on occasion, the mediation of a third reviewer (D.L.L.) was required to decide the final inclusion or exclusion of some of the documents due to the absence of agreement between the first two reviewers.

Lastly, a single reviewer (M.R.B.) performed the full data extraction of the documents finally selected in the second evaluation. A continuation of the initial Microsoft Word format data collection form was created to take note of the general information described in the study; both its identification (ID study or report) and its main characteristics (type of participants, interventions, results, etc.) again centered on compliance with the eligibility criteria defined in the review.

The Microsoft Word forms were based on the adaptation and translation of the current model conceived by The Cochrane Collaboration for RCTs [13].

## 3. Results

**Figure 1:**
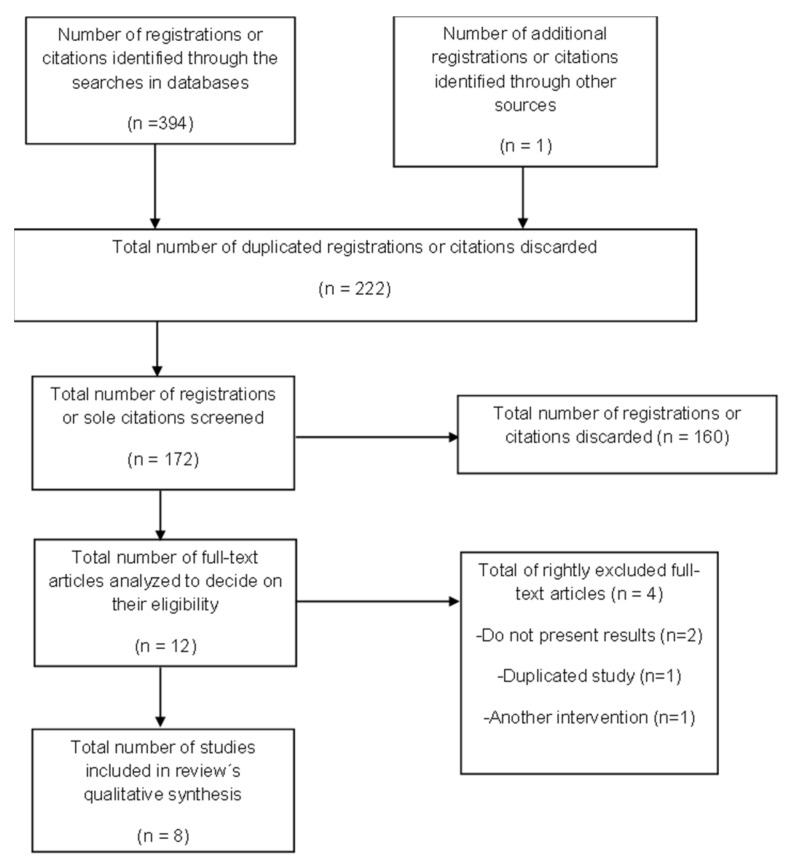
Distinct phases of the selection procedure of the studies involved in the review.

The distinct phases of the selection procedure of the studies definitively included in the review are presented in Figure 1, for which a flow diagram was utilized, based on the one established by the PRISMA declaration [13,14,15].

The design of the PICO question was based on the study selection criteria [13,16,17] (Table 1).

A synopsis of the data extracted related to the most important characteristics of the main parts of each study is presented in Table 2.

Of the eight studies included, two were RCTs [8,16], and the other six were non-controlled clinical trials [1,6,9,10,18,19]. The full number of participants in all the studies was 218 (102 men and 96 women; two studies did not specify the participants’ genders). The age ranged from 64.73 ± 7.66 to 85 years old. All the studies were conducted on adults. Interventions used were textured insoles [1,6,8,16,17,18], footwear modification [10], and habitual footwear [9]. There were nine types of comparisons used: textured insole vs. basal [18], textured insoles vs. conventional insoles [1,6,8,16,18], footwear modification vs. basal [10], habitual footwear vs. barefoot [9]. These outcomes were evaluated in the different studies: gait parameters [1,8,10,16], balance [6,17], and plantar sensation [8,16].

To better present the analysis of the studies, they were categorized according to the interventions used: textured insoles, footwear modification, and habitual footwear.

There were six studies regarding the effect of textured insoles in Parkinson’s patients [1,6,8,16,17,18].

Jenkins et al. 2009 [1] concluded there was an improvement of the gait pattern and dynamic stability. These produced an important rise in single-limb support time at the time of the initial ground contact, with the muscle activation sequence of the tibialis anterior being normalized.

In addition, Robb and Perry [18] observed that the use of textured insoles improved dynamic stability. This treatment modified the mass-base center of the support relationship. This study also showed benefits in turning performance.

Qui et al. 2013 [6] showed that this type of treatment improved postural stability, and decreased medial-lateral sway and standard deviation. In a study involving only two patients, Gaigher et al. [17] came to the same conclusion. Textured insoles improved postural stability in the most severe patients.

However, in Lirani-Silva et al. [8] there were no immediate benefits on gait with the use of textured insoles. These same authors [16] evaluated the effects one-week after the intervention and then after a one-week follow-up period. For one week, this insole amended stride length and the plantar sensation; however, only benefits in the plantar sensation were conserved after the follow-up period.

According to these studies, textured insoles would have a beneficial effect on patients with Parkinson´s disease, improving stability, plantar sensation, and gait parameters.

Regarding modifications in footwear, only one clinical case has been published by Mathieson et al. [10], describing the effect of applying an external forefoot sagittal plane wedge to raise the toes off the ground. This analysis revealed augmented step length, improved foot-strike position with the ankle nearer to a neutral to marginally dorsiflexed position, and longer midstance phase with a longer heel contact period, contrasting with the rapid unloading that distinguishes Parkinson’s gait. The author hypothesized that this footwear modification could have a role to play in the treatment of gait dysfunction in Parkinson’s disease.

In regard to the influence of wearing regular footwear compared with walking barefoot, the study carried out by Pereira et al. [9] indicated an undesirable effect of footwear on gait spatiotemporal parameters in healthy people and those with PD. PD patients showed reduced time in the swing phase and had an augmented step width variability, duration, and step width length with footwear and higher step velocity asymmetry.

Although there were very few studies on the influence of footwear in these patients, there are indications that regular footwear could have a negative influence and that modifications in footwear could be useful in their treatment.

## 4. Discussion

Lower limb problems (gait, plantar sensation, and deformities) are one of the main characteristics of PD patients [3,6,7]. Postural and motor deficiencies are among the most incapacitating symptoms for patients with PD, disturbing their quality of life [19]. These conditions respond poorly to pharmacological treatment, so it is important to consider new actions and approaches [12]. The objective of this systematic review was to determine the effect of different types of insoles and footwear in PD patients. Eight studies fulfilled the inclusion criteria and were incorporated in the review for analysis. Due to the heterogeneity detected among the interventions implemented and the use of diverse instruments for the assessment of the results, it was decided to implement a qualitative synthesis of the outcomes.

It was detected that the studies involved could be assembled into three groups according to the intervention: textured insoles [1,6,8,16,17,18], footwear modification [10], and habitual footwear [9].

Textured insoles are a type of treatment that has been studied in the most depth in these patients. Some investigations concluded that the effect of these treatments was positive for PD patients.

According to these studies, textured insoles would have an immediate beneficial effect on patients with Parkinson´s disease, improving stability and gait parameters.

Jenkins et al. 2009 [1] and Robb and Perry [19] concluded there was an improvement in dynamic stability. Jenkins et al. 2009 [1] concluded there was an improvement in the gait pattern.

Qui et al. 2013 [6] and Gaigher et al. [17] demonstrated that this type of treatment improved postural stability.

However, for Lirani-Silva et al. [8], with the use of textured insoles there were no immediate benefits on gait. These same authors [16] showed the effects one week after the intervention and after a one-week follow-up period. For one week, this insole amended the plantar sensation and gait pattern; however, only the improvement in the plantar sensation was preserved after the follow-up period. This could be due to the brief treatment period, only one week. It would be necessary to test the long-term effect of this type of treatment.

Although these studies proved that textured insoles had a beneficial effect on PD patients, only the investigations by Lirani-Silva et al. [8,16] were RCTs, the others being non-controlled clinical trials. Only Lirani-Silva et al. 2017 [16] checked the efficacy of the treatment after a follow-up period. Thus, to assess the beneficial effect of textured insoles, it would be necessary to carry out RCTs with a follow-up period.

We believe that the positive effects of this type of insole are due to plantar stimulation. Other treatments, such as plantar foot stimulation, had a positive effect on stability and gait parameters, as demonstrated by Brognara et al. in their study [2].

These variations may lead to improvement in the plantar sensation, stability, and gait parameters. It could be a convenient treatment strategy for upgrading gait quality and reducing the risk of falls in individuals with PD.

Regarding modifications in footwear, only one clinical case was published by Mathieson et al. [10], describing the effect of the application of an external forefoot sagittal plane wedge on raising the toes off the ground. The author hypothesized that this footwear modification could have a role in the treatment of gait dysfunction in PD. However, this type of intervention would not be recommended for most Parkinson’s patients because of the high prevalence of equine foot in these patients—which is 71.7% according to an observational study conducted in 2021—which would hinder the efficacy and compliance with this treatment [12].

As to the influence of wearing regular footwear compared with walking barefoot, the study carried out by Pereira et al. [9] showed a negative effect of footwear on gait spatiotemporal parameters in people with PD and healthy people. We believe that the effect on the gait parameters is due to the extra weight given by the footwear since footwear reduces peripheral feedback from the feet. The use of shoes decreases the flight phase and makes the gait more unstable and therefore more variable.

Regarding the influence of footwear on gait pattern in PD patients, modifications in footwear could have a positive effect. However, the effect of habitual footwear could be negative. According to the study by Novo-Trillo et al., a large proportion of participants with PD wear inappropriate footwear (in width, length, or both). Despite the influence that footwear has on walking and the great variability of footwear and its modifications, there are only two studies on this in PD patients. Further research would be necessary to demonstrate the effect of different types of footwear as well as their modifications in these patients: RCTs with a follow-up period. Due to the high prevalence of gait problems in these patients and the importance of footwear, it would be necessary to determine the recommended characteristics of footwear to minimize their negative effects and maximize their positive effects.

Together with dopamine-mimetic treatment, non-pharmacological treatment, like different types of insoles and shoes, could play a very important role in blocking motor symptoms, mainly for symptoms less receptive to pharmacological therapy since these symptoms tend to not respond satisfactorily to traditional therapy.

Authors should discuss the results and how they can be interpreted in the context of previous studies and their own working hypotheses. The findings and their implications should be discussed in the broadest context possible. Future research directions may also be highlighted.

Due to the small number of available RCTs, it was decided to expand the inclusion criteria by accepting any type of experimental study (randomized controlled clinical trials, non-randomized controlled clinical trials, non-controlled clinical trials, or crossover clinical trials). Of the eight studies included, two were RCTs [8,16], and the other six were non-controlled clinical trials [1,6,9,10,17,18]. The two RCTs were about investigated the effect of different textured insoles, and only Linari-Silva et al. [16] proved efficacy after a follow-up period.

Despite the high percentage of patients with biomechanical problems such as claw toes and clubfoot [5], we emphasize that no research on foot orthoses (custom-made or prefabricated) in PD patients has been found. However, the effectiveness of foot orthoses has been demonstrated for other neurodegenerative disorders [20,21,22].

**Figure 2:**
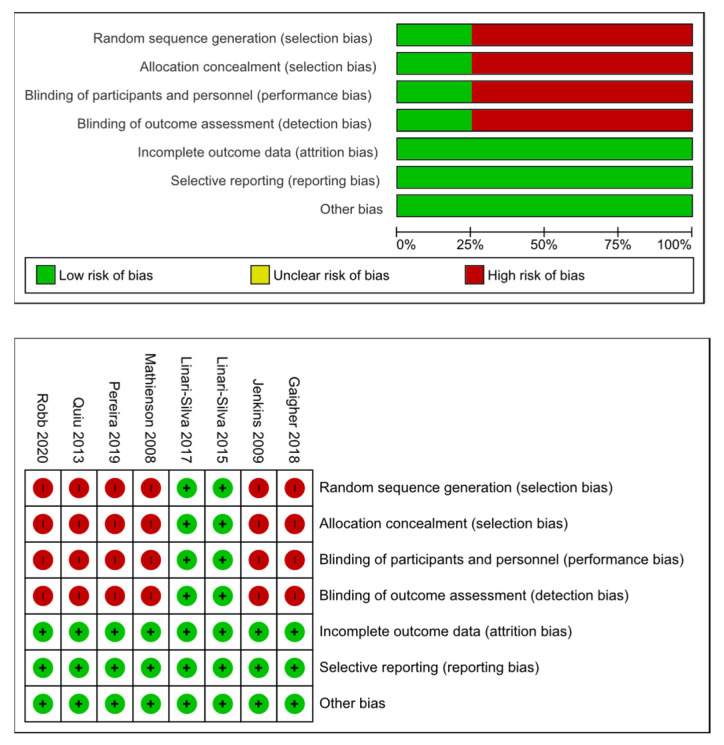
Risk of bias in the studies included. Green: Low risk of bias. Red: High risk of bias.

Several limitations have been found in this review. The total number of participants in all the studies was 218 (and two studies only included 2 participants). The number is very small; thus, it would be necessary to conduct further studies with increased number of participants In the different investigations, the disease stage and the additional treatment that patients received were not recorded. These factors could modify the effect of the interventions; therefore, they should be considered in the design of future studies. We would also recommend to record “intervention time” as another very important variable. In most studies, the effect of the different interventions was analyzed immediately, and the maximum intervention time was 5 weeks. As shown in Figure 2, 75% of the studies had a high risk of bias in four of the analyzed bias categories. This further complicates obtaining reliable results. To demonstrate the efficacy of these types of treatments in Parkinson’s patients, it would be necessary to conduct RCTs with a follow-up period.

## 5. Conclusions

According to the data available from this systematic review, the most important conclusion is that more controlled studies are needed in this research field. There are indications to suggest that textured insoles have positive effects on the gait parameters, stability, and plantar sensation in PD patients. RCTs with a follow-up period would be necessary to demonstrate the efficacy of other types of insoles (footwear modifications, habitual footwear, and foot orthoses).

## Figures and Tables

**Table 1:** Elements of the PICO question.

**P**	Participants	Study population	Parkinson´s patients
**I**	Intervention	Interesting intervention	Insole and/or footwear
**C**	Comparison	Comparison used	Any
**O**	Outcomes	Results obtained	Any
**S**	Study design	Type of study	Any type of experimental studies

**Table 2:** Main characteristics of the studies included.

	Participants	Men	Women	Average Age (Years)	Type of Treatment	Study Duration	Results Measured
Gaigher 2018	2	2		85	Textured insoles (thermoformed soles covered with textured material)	Two weeks	Postural stability
Jenkins 2009	40 Parkinson’s patients 40 control group	39	41	64.73 ± 7.66 65.40 ± 8.01	Textured insoles (facilitatory (ribbed) insole) Conventional (flat) insoles	Immediate effect	Gait parameters Dynamic stability
Lirari-Silva 2015	19 Parkinson’s patients 19 control group	20	18	71.84 ± 6.64 71.73 ± 6.64	Conventional insoles Textured insoles (with half-spheres) Textured insoles (with a raised ridge around the foot perimeter)	Immediate effect	Gait parameters Plantar sensation
Lirari-Silva 2017	10 experimental group 9 control group			70.4 ± 6.87 72 ± 6.2	Textured insoles (with half-spheres) Conventional insoles	One week	Gait parameters Plantar sensation
Mathieson 2008	1 Parkinson’s patient				Footwear modification	Immediate effect	Gait parameters
Pereira 2019	16 Parkinson’s patients 15 control group	13	18	69.19 ± 7.25 70.80 ± 5.51	Habitual footwear	Immediate effect	Gait parameters
Qiu 2013	20 Parkinson’s patients 20 control group	26	14	65 ± 9 69 ± 5	Smooth insoles Textured insoles	Immediate effect	Static balance
Robb 2020	7 Parkinson’s patients	2	5	71.5 ± 8.3	Conventional insoles Textured insoles	Five weeks	Dynamic balance Gait parameters

The risk of bias in the studies included is shown in Figure 2.

## Supplementary Materials

The following are available online at https://www.mdpi.com/article/10.3390/jpm11111136/s1, Search strategy.
