# Supplementary material for: Effect of Foot Orthoses and Shoes in Parkinson’s Disease Patients: A PRISMA Systematic Review
Source: J Pers Med. 2021 Nov 2;11(11):1136. doi: 10.3390/jpm11111136 (PMC8621527; doi:10.3390/jpm11111136)
Supplement: Supplementary file 1 [file jpm-11-01136-s001.zip › jpm-1429709-supplementary.pdf]

Supplementary: Search strategy

Pubmed

-("Parkinson Disease"[Mesh]) AND "Foot Orthoses"[Mesh]

-("Parkinson Disease"[Mesh]) AND "Shoes"[Mesh]

-foot orthoses AND parkinson

-insoles AND parkinson

-shoes AND parkinson

-foot wear AND parkinson

Scopus

- "Parkinson" AND "insole"

- "Parkinson" AND "foot orthoses"

- "shoes" AND "parkinson"

- “foot wear” AND “parkinson”

Cochrane Reviews

-"parkinson" AND "foot wear"

-“insoles” AND “parkinson”

- "shoes" AND "parkinson"

- "foot orthoses" AND "parkinson"

Dialnet

- plantillas AND parkinson

-ortesis plantares AND parkinson

- calzado AND parkinson

-zapato AND parkinson

-zapato AND parkinson

Web of Science

- foot orthoses AND parkinson

-foot wear AND parkinson

-insole AND parkinson

-shoes AND parkinson
